# An Automatic Refolding Apparatus for Preparative-Scale Protein Production

**DOI:** 10.1371/journal.pone.0045891

**Published:** 2012-09-27

**Authors:** Yanye Feng, Ming Zhang, Linlin Zhang, Ting Zhang, Jianfeng Ding, Yingping Zhuang, Xiaoning Wang, Zhong Yang

**Affiliations:** 1 State Key Laboratory of Bioreactor Engineering, School of Biotechnology, East China University of Science and Technology, Shanghai, China; 2 State Key Laboratory of Genetic Engineering, Department of Microbiology, School of Life Sciences, Fudan University, Shanghai, China; 3 Department of R&D, Novoprotein Scientific Inc, Shanghai, China; Aligarh Muslim University, India

## Abstract

Protein refolding is an important process to recover active recombinant proteins from inclusion bodies. Refolding by simple dilution, dialysis and on-column refolding methods are the most common techniques reported in the literature. However, the refolding process is time-consuming and laborious due to the variability of the behavior of each protein and requires a great deal of trial-and-error to achieve success. Hence, there is a need for automation to make the whole process as convenient as possible. In this study, we invented an automatic apparatus that integrated three refolding techniques: varying dilution, dialysis and on-column refolding. We demonstrated the effectiveness of this technology by varying the flow rates of the dilution buffer into the denatured protein and testing different refolding methods. We carried out different refolding methods on this apparatus: a combination of dilution and dialysis for human stromal cell-derived factor 1 (SDF-1/CXCL12) and thioredoxin fused-human artemin protein (Trx-ARTN); dilution refolding for thioredoxin fused-human insulin-like growth factor I protein (Trx-IGF1) and enhanced fluorescent protein (EGFP); and on-column refolding for bovine serum albumin (BSA). The protein refolding processes of these five proteins were preliminarily optimized using the slowly descending denaturants (or additives) method. Using this strategy of decreasing denaturants concentration, the efficiency of protein refolding was found to produce higher quantities of native protein. The standard refolding apparatus configuration can support different operations for different applications; it is not limited to simple dilution, dialysis and on-column refolding techniques. Refolding by slowly decreasing denaturants concentration, followed by concentration or purification on-column, may be a useful strategy for rapid and efficient recovery of active proteins from inclusion bodies. An automatic refolding apparatus employing this flexible strategy may provide a powerful tool for preparative scale protein production.

## Introduction

An explosion in the field of structural genomics and protein expression has greatly increased our knowledge of how to manipulate proteins [1], [2]. One of the most attractive means of producing recombinant proteins utilizes genetically modified *E. coli*, due to their low cost, high productivity, and ease of use [3]. However, a substantial hindrance to these efforts is the formation of insoluble inclusion bodies. Nevertheless, it is possible to denature and refold the insoluble proteins, with an increasing number of refolding approaches [4]. The procedures for refolding proteins from inclusion bodies consisted of 1) solubilizing inclusion bodies with denaturing agents, and 2) refolding proteins through the removal of denaturants. However, the refolding of proteins from inclusion bodies must still be largely performed with a series of trial-and-error renaturation experiments.

According to the Refold Home dataset, the most common methods reported in the literature regarding protein refolding include dilution, step-wise dialysis and on-column techniques [4]. Due to its simplicity, refolding by simple dilution has been the most common method used in industry and academia [5], [6] for large-scale protein production [7]. However, in the one-step dilution refolding process, the denatured protein is refolded rapidly and has insufficient time to produce a homogeneous population, often leading to the formation of aggregates [8]. In a step-wise dialysis refolding process, the denatured, unfolded and fully reduced proteins containing a high concentration of denaturant are placed in a dialysis bag. The dialysis bag containing the denatured protein is then submerged successively into dialysis buffer with a high, middle and low concentration of denaturant. Although the step-wise dialysis is a useful method for protein refolding, the solvent composition in the dialysis bag at each denaturant concentration could not be manipulated during dialysis. For proteins without established protocols, unsuitable solvent compositions can result in massive protein aggregation using a step-wise dialysis method. To prevent such unspecific aggregation, on-column refolding made use of the specific interactions of the protein [7]. Many recombinant proteins were successfully recovered from inclusion bodies using the on-column refolding method [9], [10]. However, the refolding yield decreased with the increase of protein loaded onto the column [10]. Because these refolding methods have both advantages and disadvantages, we wanted to combine two or three of them together to complement each other to achieve a better outcome.

The recovery of active recombinant proteins from inclusion bodies is also a time consuming and laborious process, including multiple steps such as solution preparation, protein concentration, purification and trials of refolding techniques. Due to the inconsistencies and difficulties of the protein refolding process, there is a need for an automated large-scale refolding apparatus that integrated dilution, dialysis and on-column refolding, thereby reducing the need for experimenter’s time.

An effective refolding apparatus system configuration should support multiple operations for different applications, including refolding condition variables, such as total protein concentration, denaturants concentration, refolding time, fluid path and refolding methods. The refolding apparatus can be set and maintained throughout the refolding process by adjusting the flow rates of the buffer, the denatured protein suspension, or the fluid path. In this study, we integrated various refolding approaches followed by protein concentration and purification on column. Different methods were carried out to evaluate the performance of this refolding apparatus: a combination of dilution and dialysis for human stromal cell-derived factor 1 (SDF-1/CXCL12) and thioredoxin-human artemin fusion protein (Trx-ARTN); dilution refolding for thioredoxin-human insulin-like growth factor I fusion protein (Trx-IGF1) and enhanced fluorescent protein (EGFP); and on-column refolding for BSA. The refolding processes of these five proteins were preliminary optimized using a strategy of slowly descending denaturant concentration.

## Results

### Design of Refolding Apparatus

**Figure 1:**
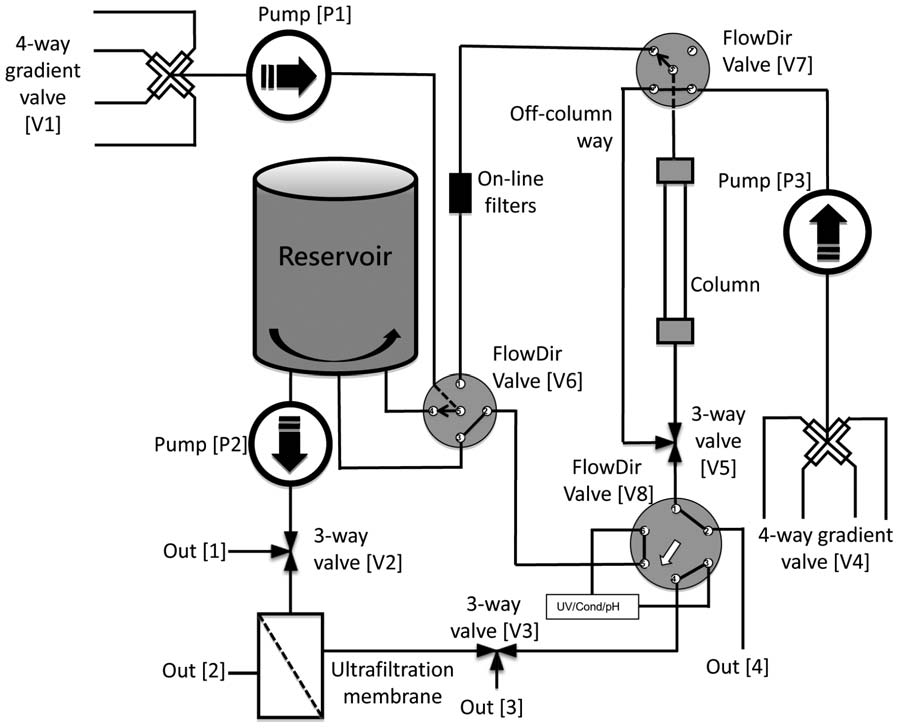
Schematic representation of the refolding apparatus.

The refolding apparatus consisted of a compact separation unit and a control system unit with a laptop computer running the system control software. The compact separation unit utilized three refolding techniques that were carried out, respectively, by a dilution module, an ultrafiltration module and an on-column refolding module. Figure 1 shows the location of the modules and components of the separation unit.

In the fluid path of the refolding apparatus, the three refolding modules were coupled with a refolding reservoir. The dilution module consisted of a 4-channel gradient valve (V1), a pump (P1) and a refolding reservoir. Using pump (P1) and 4-channel gradient valve (V1), the denatured, unfolded and fully reduced protein samples in a concentrated denaturant solution were directly placed into the refolding reservoir that contained a large volume of buffer.

**Figure 2:**
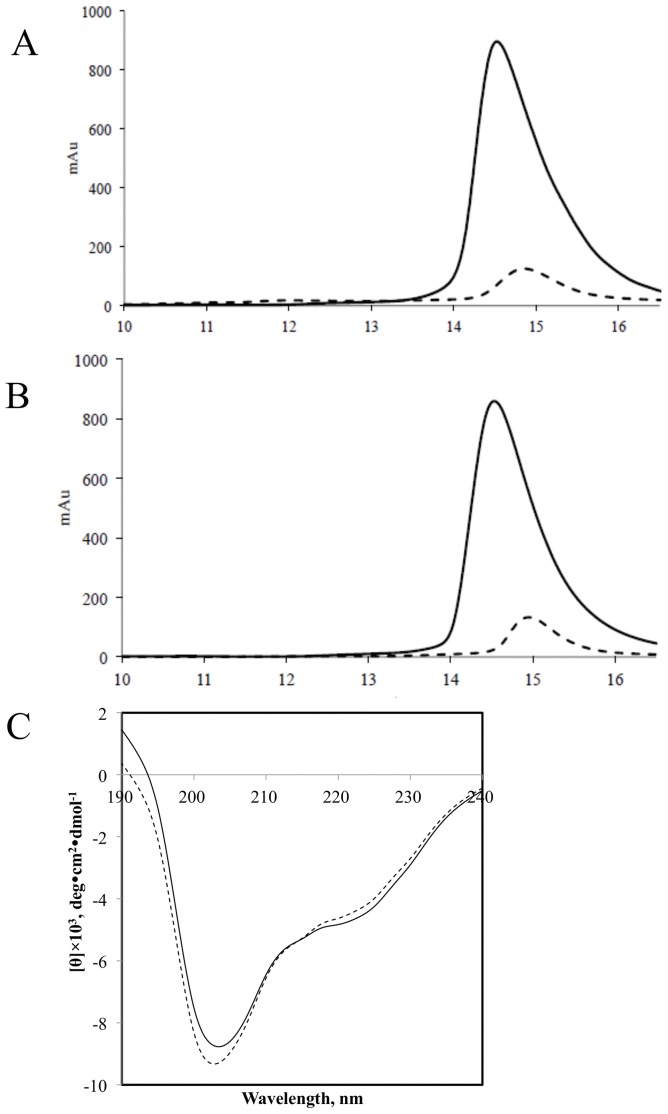
Analysis of SDF-1/CXCL12. Size exclusion chromatography using a Superdex-75 column for SDF-1/CXCL12 produced by a combination of dilution and dialysis refolding technique (A) and a combination of reverse-dilution and dialysis refolding technique (B). SDF-1/CXCL12 eluted as a single peak for both high-concentration injected mass (solid line) and low-high-concentration injected mass (dotted line). (C) A comparison of CD spectra of SDF-1/CXCL12 between a combination of dilution and dialysis refolding technique (dotted line) and a combination of reverse-dilution and dialysis refolding technique (solid line). Molecular weight values were determined by column calibration with protein standards (Figure 5S). The retention volumes for aprotinin (Mr 6,512 Da) and ribonuclease (Mr 13,683Da) on the Superdex-75 10/300 HR column were 15.3 ml and 13.1 ml, respectively.

The ultrafiltration module was attached to the refolding reservoir to aid in the refolding by dialysis. The ultrafiltration module contained two 3-way valves (V2 and V3), a pump (P2) and an ultrafiltration device. Each 3-way valve was assembled using two 2-way valves. In the dialysis refolding, denatured, unfolded and fully reduced protein samples in concentrated denaturant solution were held in the refolding reservoir. A renaturation buffer was continuously fed into the refolding reservoir with pump (P1); simultaneously, the denaturants were continuously filtered and removed using an ultrafiltration membrane. The retentate with proteins was returned to the refolding reservoir. Conventionally, the dialysis bag that contained the denatured protein was immersed in a large volume of refolding solvent in a large reservoir. In this study, the denatured protein was held in the reservoir, not a dialysis bag, so refolding efficiency was controlled easily by both adding solvent additives directly and manipulating the folding pathway, as previously reported [11], [12]. Additionally, the dialysis module could be used to concentrate the protein solution. In general, the change in concentration of denaturants in the reservoir over time was expressed as:
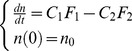
(1)


In Equation 1, n is the molar mass of denaturants in the refolding reservoir, C_1_ is the concentration of denaturants in the feed buffer added into the refolding reservoir, F_1_ is the flow rate of the feed buffer added into the refolding reservoir, F_2_ is the flow rate of the removed filtrate, C_2_ is the concentration of denaturants in the removed filtrate as described in 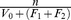, and V_0_ is the initial volume of buffer in the refolding reservoir. If C_1_ = 0 and F_1_ = F_2_, the volume of the buffer in the refolding reservoir was constant, and the change in concentration of denaturants (i.e., 8 M urea) over time can be calculated as follows:
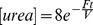
(2)


In Equation 2, F is the feed flow rate or outlet filtrate, and V is the volume of buffer in the refolding reservoir. Here, the F/V value weighed the extent of the decreasing denaturant concentration. Based on Eq. 2, as the ratio between the feed flow rate and the volume of buffer in the refolding reservoir increases, the rate of decreasing denaturant concentration increases. The rates of pumping-in and out determine the gradient of descending denaturant concentration. Therefore, the advantage of a dialysis unit is that the solvent composition in the reservoir can be manipulated at each denaturant concentration. The dialysis unit was a convenient tool to manipulate and monitor some important variables such as denaturant concentration, the change rate of denaturant concentration, ionic strength and redox environments. However, it is hard to determine in real-time the solvent composition when the protein solution was held in a dialysis bag.

To expand the versatility of the apparatus, components for refolding on-column or on-line column capture of refolded proteins were added to the apparatus. These improvements included three FlowDir valves (V6, V7 and V8), on-line filters, a 4-channel gradient valve (V4), a Pump (P3), one 2-way valve (V5), a conductivity/UV/pH detector and a series of packed columns. To remove the aggregates formed in the reservoir, on-line filters containing 10 µm and 1 µm filter membranes were added. These filters protected the protein chromatography columns when the soluble protein solution from the reservoir was loaded onto the column. Four different solvents could be chosen using the 4-channel gradient valve (V4), which could serve as the protein purification system. The FlowDir valves (V6 and V7) were five-way valves; at a given setting, two adjacent channels were connected and an axial channel was connected with one of the remaining two channels. The FlowDir valve (V8) was a six-way valve, in which every two adjacent channels were connected. The FlowDir valve (V6) linked pump (P1), reservoir, FlowDir valve (V7) and FlowDir valve (V8). The FlowDir valve (V6) could switch between four positions: (i) linking pump (P1) and the reservoir, or FlowDir valve (V8) and the reservoir; (ii) linking pump (P1) and FlowDir valve (V7); (iii) linking pump (P1) and FlowDir valve (V8); (iv) linking FlowDir valve (V8) and FlowDir valve (V7), or pump (P1) and the reservoir (see Figure S1). The FlowDir valve (V7) linked pump (P3), FlowDir valve (V6), an off-column port and a column, and could switch between three positions: (i) linking FlowDir valve (V6) and the column, or pump (P3) and the off-column way; (ii) linking FlowDir valve (V6) and the off-column way; (iii) linking pump (P3) and the column (see Figure S2). The FlowDir valve (V8) had two switch positions, the detector connected with the column or FlowDir valve (V6) (see Figure S3).

**Table 1:** Purification process of SDF-1/CXCL12.

	Volume (ml)		Concentration (mg/ml)^a^		Total protein mass (mg)		Purity (%)^b^	
Steps	P1	P2	P1	P2	P1	P2	P1	P2
Solubilization	10	10	3	3	30	30	∼60	∼60
Refolding & Dialysis*	500	500	0.037	0.043	18.5	21.5	NA	NA
CM-sepharose FF*	11	17	0.5	0.62	5.5	10.5	∼89	∼92
Superdex-75 10/300	2.5	1.5	1.85	5.8	4.6	8.7	∼95	∼96

a Protein concentration was determined by Bradford assay.

b Protein purity was determined by SDS-PAGE with Coomassie Blue Staining.

P1, Purification process of SDF-1/CXCL12 by dilution and dialysis combination refolding method.

P2, Purification process of SDF-1/CXCL12 by reverse-dilution and dialysis combination refolding method.

* , Steps were carried on the refolding apparatus.

NA, Not Available.

### An Overview of Protein Refolding Methods of Five Proteins

We developed protocols to refold and oxidize SDF-1/CXCL12, Trx-ARTN, and Trx-IGF1 from bacterially expressed inclusion bodies in previous study, whereas the protein refolding efficiency was low (unpublished data). Therefore, we would try to improve the refolding efficiency of these proteins using different refolding techniques on this apparatus. In addition, we chose BSA and EGFP to compare protein refolding efficiency with previously studies. Furthermore, these five proteins have different molecular weight (from 8.9 kDa to 66.4 kDa), pI (acidic or basic) and folded states (monomer, dimer, and self-associated), and used different denaturants and refolding techniques. In summary, these five proteins can have certain representativeness and are used to demonstrate the advantage and generality of this apparatus (see Table S1, An overview of information of five proteins).

First, the widely used simple dilution, dialysis and on-column refolding methods are to test with several proteins: a combination of dilution and dialysis for SDF-1/CXCL12 and Trx-ARTN; dilution refolding for Trx-IGF1 and EGFP; and on-column refolding for BSA. In the refolding process of SDF-1/CXCL12 or Trx-ARTN, GdnHCl denatured proteins were diluted into a refolding buffer containing L-Arg followed by dialysis to remove L-Arg. In the refolding process of Trx-IGF1 or EGFP, denatured proteins dissolved in GdnHCl or N-lauroylsaccosine were directly diluted into refolding buffer. For the refolding of BSA, urea denatured BSA was loaded on ion-exchange resin and incubated in a refolding buffer. In addition, after refolding, the solutions of SDF-1/CXCL12, Trx-ARTN, and Trx-IGF1 were directly loaded on resin and separated.

Previously, we reported an efficient method to recover active protein from inclusion bodies, called the two-step-denaturing and refolding method (TDR) [13]. In the refolding of the catalytic domain of human macrophage metalloelastase (MMP-12), we used continuous dialysis to generate a slowly decreasing concentration gradient of denaturant to allow the protein to refold. We found that the slowly decreasing concentration of denaturant method was a sound strategy to recover active protein from denatured protein, which assumes sequential structure formation occurring in parallel with descending concentration of denaturant agent [8], [14]. In this study, the slowly decreasing denaturants concentration procedure was performed on the apparatus by means of a reverse-dilution method (for SDF-1/CXCL12, Trx-ARTN and Trx-IGF1) or a continuous dialysis method (for BSA and EGFP). In the reverse-dilution method, the refolding buffer was slowly added to the denatured proteins, which generated a linear concentration gradient for decreasing denaturants and increasing additives. In the continuous dialysis method, as described in the section “design of refolding apparatus”, a non-linear concentration gradient of decreasing denaturants (or increasing additives) was generated to allow the protein to refold through pumping out the denaturing buffer and pumping in refolding buffer. Furthermore, after refolding, the soluble protein solution was directly loaded on resin and separated.

To test the quality and efficiency of an optimized refolding process and to compare it with a previous refolding process, we analyzed protein refolding yield and their folding properties. The protein refolding yield was the yield of the total soluble protein compared with the starting amount of denatured proteins. Protein folding properties were used to estimate protein refolding recovery. SDF-1/CXCL12 (Mr, 8.9 kDa; pI, 10.5) is a small cytokine belonging to the chemokine family, which has 4 conserved cysteines forming 2 disulfide bonds [15], [16], [17]. It was previously described as a monomer at low concentrations and a dimer at high protein concentrations, using size-exclusion chromatography and circular dichroism to monitor its biophysical properties [17]. Size-exclusion chromatography analysis can indicate whether the chemokine self-associates, whereas circular dichroism spectrum provide information regarding protein structure. Trx-ARTN (Mr, 26 kDa; pI, 9.0) is a fusion protein of thioredoxin and the human artemin protein, which is a disulfide-linked homodimer and a neurotrophic growth factor of the GDNF ligand family that signals through the specific GFRα-3 coreceptor/cRet tyrosine kinase-mediated signaling cascade [18], [19]. Size-exclusion chromatography and non-reduced SDS-PAGE analysis were used to monitor the biophysical properties of Trx-ARTN. Size-exclusion chromatography demonstrated that soluble Trx-ARTN has the correct molecular weight corresponding to a dimer. Non-reduced SDS-PAGE analysis showed that the intermolecular disulphide bridge was formed in the homodimer [20], [21]. Trx-IGF1 (Mr, 25 kDa; pI, 5.6) is a fusion protein of thioredoxin and the human insulin-like growth factor 1. IGF1 is a monomeric hormone similar to insulin in molecular structure [22], [23], [24]. In the refolding of Trx-IGF1, the recovery of monomer was very low due to protein aggregation. Size-exclusion chromatography demonstrated that refolded Trx-IGF1 has the correct molecular weight of the monomer, not aggregates. BSA (Mr, 66 kDa; pI, 5.6) was used as a model protein for refolding and oxido-shuffling on an ion-exchange column [10]. RP-HPLC and circular dichroism were used to compare physical characteristics of refolded BSA against the native BSA as reported methods [10]. EGFP (Mr, 27 kDa; pI, 5.7) was chosen as a model protein, because it could be produced in both soluble form and insoluble inclusion bodies in the *E. coli* [13], [25]. The refolding efficiency of EGFP can be easily monitored using fluorescent spectrometry [26].

### Refolding of SDF-1/CXCL12

We developed a protocol to refold and oxidize SDF-1/CXCL12 from bacterially expressed inclusion bodies, which were solubilized using denaturing buffer containing 6 M guanidine hydrochloride. The denatured proteins were slowly added into the refolding buffer containing 0.75 M L-Arg. After 24 h, the refolded proteins were dialyzed against an equilibrium buffer and purified using a CM sepharose resin. Finally, the CM sepharose eluate was analyzed using a Superdex-75 column. The chemokine SDF-1/CXCL12 is a monomer at low protein concentrations, but at concentrations above 200 µM, the observed mass of SDF-1/CXCL12 corresponded to a dimer [17].

The refolding procedure on the refolding apparatus can be divided into three parts. In the initial preparation step, ddH_2_O and refolding buffer were used to rinse the 4-way gradient valve (V1) and pump (P1) into the reservoir through the Dirflow valve (V6) and then pumped out by pump (P2) through 3-way valve (V2). Then, a defined amount of refolding buffer was introduced into the reservoir, and denatured SDF-1/CXCL12 was slowly added into the refolding buffer. Before the end of the refolding time in the solution containing L-Arg, equilibrium buffer was used to rinse 4-way gradient valve (V1), pump (P1), the detector (optional) and the ultrafiltration membrane through Dirflow valve (V6) and Dirflow valve (V8). Afterward, equilibrium buffer was continuously fed into the reservoir by pump (P1). At the same time, the SDF-1/CXCL12 solution was pumped through the ultrafiltration membrane by pump (P2) and returned to the reservoir through Dirflow valve (V8) and Dirflow valve (V6). After 12 h of dialysis, equilibrium buffer was used to rinse 4-way gradient valve (V1), pump (P1) and the detector through Dirflow valve (V6), Dirflow valve (V7), Dirflow valve (V8), and the off-column tubing. Then, equilibrium buffer was introduced into the column to equilibrate the column by adjusting Dirflow valve (V7). Next, soluble SDF-1/CXCL12 solution can be directly loaded onto the column by pump (P2) through Dirflow valve (V8), Dirflow valve (V6), Dirflow valve (V7) and the detector. The refolded SDF-1/CXCL12 protein was eluted with a linear gradient from 4-way gradient valve (V1). In the last step, the washing buffer was pumped into the system to wash the entire system. An animation of the entire refolding process of SDF-1/CXCL12 was supplied in the supporting information (Video S1: CXCL12 refolding process of dilution and dialysis combination.mp4).

Using this refolding apparatus, from 30 mg of ∼60% pure denatured proteins, 5.5 mg of ∼89% pure and soluble SDF-1/CXCL12 was successfully eluted from the CM sepharose resin. Subsequently, SDF-1/CXCL12 was further purified by size-exclusion chromatography. 4.6 mg of ∼95% pure and soluble SDF-1/CXCL12 was obtained and analyzed by Superdex-75 (Table 1). Two different protein concentrations of SDF-1/CXCL12 (1.2 and 8 mg/ml) were injected onto the Superdex-75 column. The retention volumes for SDF-1/CXCL12 were 14.86 ml for the 1.2 mg/ml injection and 14.52 ml for the 8 mg/ml injection, corresponding to 6.8 kDa and 8.0 kDa (Figure 2), respectively, which was identical to previously reported data [17]. Size exclusion chromatography analysis demonstrated that SDF-1/CXCL12 could self-associate. The CD spectrum of purified SDF-1/CXCL12 showed that the soluble protein has secondary folding containing a mixture of α-helix and β-sheet structures (Figure 2C). The shape of CD spectrum and main CD features of secondary structures was in accordance with previously reported data [17].

### Optimizing CXCL12 Refolding Process

We successfully improved the refolding yield of SDF-1/CXCL12 by slowly decreasing denaturants (or additives) concentration. The optimized refolding process used a refolding buffer that contained 0.75 M L-Arg and was slowly added into the 6 M GdnHCl denatured protein solution. The initial protein concentration was 1.0 mg/ml. This reverse-dilution method generated a linear decreasing concentration of denaturant (GdnHCl)to 0.6 M at a rate of 4.2 mM/min, a linear decreasing protein concentration to 0.1 mg/ml at a rate of 0.042 mg/h, and a linear increasing L-Arg concentration to 0.7 M at a rate of 0.53 mM/min. Otherwise the refolding process was the same as described above. Using this optimized refolding process, 10.5 mg of ∼92% pure and soluble SDF-1/CXCL12 was eluted from CM sepharose resin from 30 mg of ∼60% pure denatured proteins. The animation of the entire refolding process of SDF-1/CXCL12 was supplied in supporting information (Video S2: CXCL12 refolding process of reverse-dilution and dialysis combiantion.mp4).

Finally, 8.7 mg of ∼96% pure and soluble SDF-1/CXCL12 was obtained after size-exclusion chromatography (Table 1). After optimization, the amount of purified SDF-1/CXCL12 was almost double of that using the previous refolding process. Two different protein concentrations of SDF-1/CXCL12 (1.6 and 8 mg/ml) were also injected onto the Superdex-75 column. The retention volumes for SDF-1/CXCL12 were 14.95 ml for the 1.2 mg/ml injection and 14.53 ml for the 8 mg/ml injection, corresponding to 6.6 kDa and 7.9 kDa (Figure 2B), respectively. All SDF-1/CXCL12 proteins in both refolding processes had almost the same elution volume. Size exclusion chromatography analysis demonstrated that SDF-1/CXCL12 could self-associate. The CD analysis yielded spectra characteristic of purified SDF-1/CXCL12 in accordance with previously reported data [17].

We also tried to increase the rate of decreasing denaturants concentration to 8.4 mM/min. As a result, 7 mg of ∼91% pure and soluble SDF-1/CXCL12 was eluted from CM sepharose resin from 30 mg of ∼60% pure denatured proteins. However, a great deal of aggregation was observed during protein concentration, prior to further purification using size exclusion chromatography. Finally, only 3.6 mg of ∼96% pure and soluble SDF-1/CXCL12 was obtained after size exclusion chromatography. Perhaps, the slowly descending denaturants concentration needs to be synchronized with the protein refolding process. If the rate of decreasing of denaturants concentration was increased, it would possibly disturb the synchronization and lead to misfolded proteins, which would initiate aggregation.

### Refolding Trx-ARTN, Trx-IGF1, BSA and EGFP by Slowly Descending Denaturants Concentration

**Figure 3:**
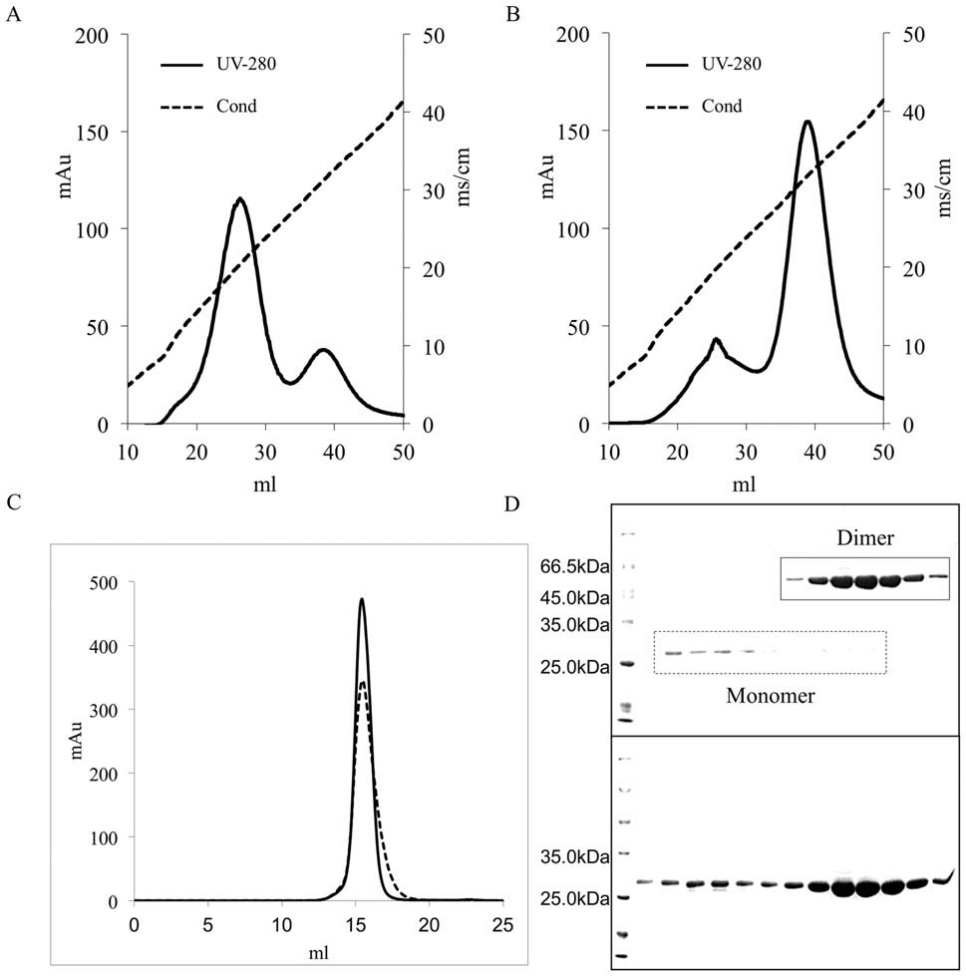
Analysis of Trx-ARTN. Cation exchange chromatography using a HiTrap CM FF column for Trx-ARTN produced by a combination of dilution and dialysis refolding technique (A) and a combination of reverse-dilution and dialysis refolding technique (B). Monomeric and dimeric Trx-ARTN proteins were eluted at low and high NaCl concentrations, respectively. Protein-containing fractions were analyzed by non-reduced SDS-PAGE (top) and reduced SDS-PAGE (bottom) to determine the molecular weight of refolded and partially folded protein (C). (D) Size-exclusion chromatography of Trx-ARTN produced by dilution and dialysis combination refolding technique (dotted line) and reverse-dilution and dialysis combination refolding technique (solid line). Molecular weight values were determined by column calibration with protein standards (Figure 6S). The retention volumes for B-lactoglobulin (Mr 35,000 Da) and BSA (Mr 67,000 Da) on the Superdex-200 10/300 HR column were 15.5 ml and 14 ml, respectively.

Trx-ARTN was expressed in *E. coli* as inclusion bodies. 5.76 mg of ∼90% pure dimeric protein from 23.7 mg of ∼80% pure denatured protein was obtained by slowly decreasing the denaturants concentration. The reverse-dilution and dialysis combination method produced almost three-fold the amount (1.4 mg) from 18 mg of ∼75% pure denatured protein by a combination of dilution and dialysis (Table 2). During the refolding and dialysis step, there were almost the same refolding yields of soluble form protein in both protein refolding processes. However, after CM sepharose resin separation, the monomeric and dimeric Trx-ARTN proteins were eluted one after the other by a linear gradient elution (Figure 3A, 3B), respectively, at low and high NaCl concentration. More dimeric Trx-ARTN was produced by slowly descending denaturants concentration than by a combination of dilution and dialysis. Dimeric Trx-ARTN samples eluted from the CM-sepharose column from both refolding methods were analyzed using the Superdex-200 column (Figure 3C). Size exclusion chromatography analysis demonstrated that two Trx-ARTN samples had almost the same elution volume of 15.4 ml corresponding to 40 kDa, which suggested that refolded Trx-ARTN had a correct molecular weight of the dimer. Non-reduced SDS-PAGE analysis showed that the intermolecular disulphide bridge was formed in the homodimer (Figure 3D). The animation of the refolding process of Trx-ARTN was the same as SDF-1/CXCL12.

**Table 2:** Purification process of Trx-ARTN.

	Volume (ml)		Concentration (mg/ml)^a^		Total protein mass (mg)		Purity (%)^b^	
Steps	P1	P2	P1	P2	P1	P2	P1	P2
Solubilization	41	50	1.1	0.64	45.1	32	∼60	∼65
Ni-column	45	37	0.49	0.64	18	23.7	∼75	∼80
Refolding & Dialysis*	750	700	0.013	0.015	9.75	11	NA	NA
CM-sepharose FF*	14	16	0.1	0.36	1.4	5.76	∼92	∼90

a Protein concentration was determined by Bradford assay.

b Protein purity was determined by SDS-PAGE with Coomassie Blue Staining.

P1, purification process of Trx-ARTN by dilution and dialysis combination refolding method.

P2, purification process of Trx-ARTN by reverse-dilution and dialysis combination refolding method.

* , Steps were carried on the refolding apparatus.

NA, Not Available.

**Figure 4:**
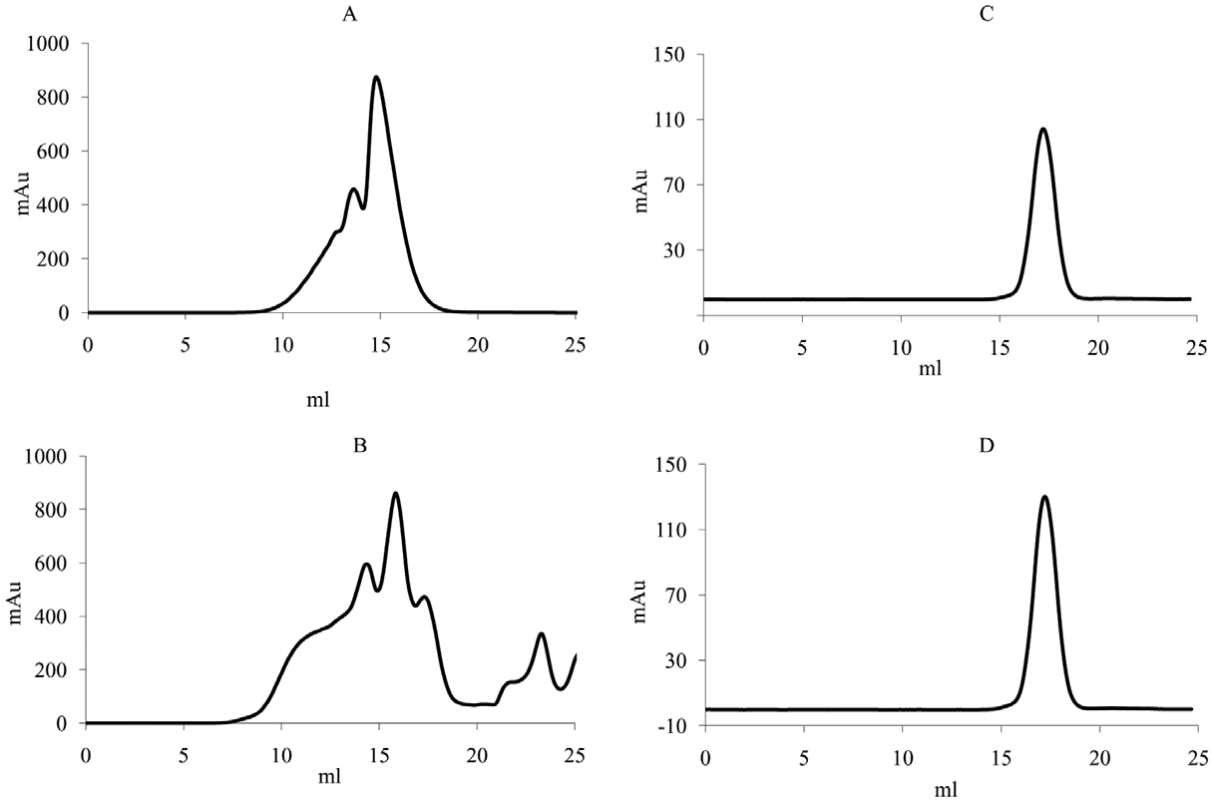
Size exclusion chromatography using a Superdex-200 column for Trx-IGF1 produced by dilution refolding technique (A, C) and reverse-dilution refolding technique (B, D). Trx-IGF1 eluted from Ni-column was purified by size exclusion chromatography (A, B). Size exclusion chromatography demonstrated that two pure Trx-IGF1 samples had almost the same elution volume (C, D).

Trx-IGF1 was expressed in *E. coli* as inclusion bodies. 2.2 mg of ∼95% pure and monomeric protein from 25 mg of ∼72% pure denatured protein was produced by slowly decreasing denaturants concentration. The reverse-dilution method produced almost two-fold more than the amount (1.0 mg) of soluble Trx-IGF1obtained from 30.1 mg of ∼70% pure denatured protein by rapid dilution. At each stage during the refolding and dialysis and Ni-column purification and concentration steps, there were almost the same refolding yields of soluble protein from both protein refolding processes (Table 3). However, the refolding yields of the monomeric protein varied dramatically between the two protein refolding processes (Figure 4A, 4B). An elution peak of monomeric protein was separated from other oligomeric proteins using reverse-dilution. But rapid dilution caused more oligomeric proteins to form. Finally, size exclusion chromatography demonstrated that two pure Trx-IGF1 samples had almost the same elution volume of 17.2 ml corresponding to 17 kDa (Figure 4C, 4D), indicating that two Trx-IGF1 samples were all monomers instead of aggregates. The animations of both refolding processes of Trx-IGF1 were supplied in the supporting information (Video S3: Trx-IGF1 refolding process of dilution.mp4; Video S4: Trx-IGF1 refolding process of reverse-dilution.mp4).

**Table 3:** Purification process of Trx-IGF1.

	Volume (ml)		Concentration (mg/ml)^a^		Total protein mass (mg)		Purity (%)^b^	
Steps	P1	P2	P1	P2	P1	P2	P1	P2
Solubilization	50	40	3.5	1.3	175	52	∼65	∼67
Ni-column	43	50	0.7	0.5	30.1	25	∼70	∼72
Refolding*	500	500	0.057	0.48	28.5	24	NA	NA
Ni-column*	44	40	0.057	0.53	25.1	21	∼87	∼86
Concentration	0.55	0.55	19.1	21.8	10.5	12	∼80	∼77
Superdex-200 10/300	2.5	3	1.46	1.0	3.6	3	∼90	∼91
Superdex-200 10/300	1.5	1.5	0.7	1.46	1.0	2.2	∼95	∼95

a Protein concentration was determined by Bradford assay.

b Protein purity was determined by SDS-PAGE with Coomassie Blue Staining.

P1, purification process of Trx-IGF1 by dilution refolding technique.

P2, purification process of Trx-IGF1 by reverse-dilution refolding technique.

* , Steps were carried on the refolding apparatus.

NA, Not Available.

**Figure 5:**
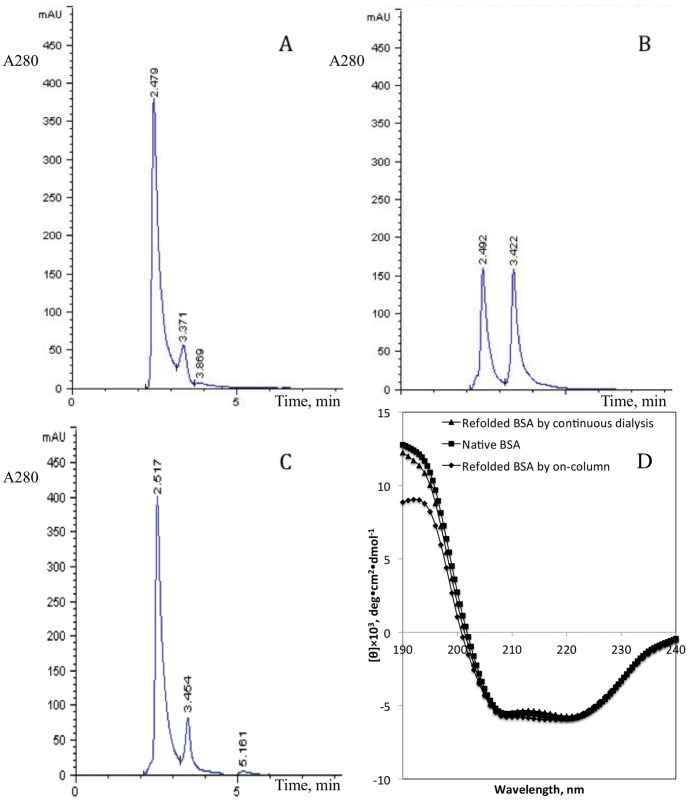
Analysis of BSA. RP-HPLC chromatograms of native BSA (A), pooled fractions eluted from an ion-exchange column within on-column refolding technique (B) and continuous dialysis refolding technique (C). (D) A comparison of CD spectra of native, on-column refolded and continuous dialysis refolded BSA under native solution condition.

We then tested the previously reported refolding procedures of BSA [10] and EGFP [27]. These two proteins were used to evaluate the performance of the continuous dialysis refolding method (Video S5: BSA refolding process of on-column.mp4; Video S6: BSA refolding process of continuous dialysis.mp4; Video S7: EGFP refolding process of dilution.mp4; Video S8: EGFP refolding process of continuous dialysis.mp4). First, the previous refolding procedures of BSA and EGFP, were carried out on this apparatus step by step. Then, continuous dialysis refolding of BSA and EGFP, using the same refolding buffer reported in the literature, were performed as described in Materials and Methods. A comparison of the refolding recovery of BSA between both refolding processes is shown in Figure 5. The on-column refolded BSA showed a similar amount of protein refolding recovery (∼50%, Figure 5B) and yield (∼60%) as reported previously when 2 mg of denatured protein loaded on a Q-sepharose fast flow column [10]. This suggested that some protein aggregated and precipitated after being desorbed from the matrix. In contrast, BSA refolded by continuous dialysis showed a higher amount of recovery (∼90%, Figure 5C) and yield (∼80%) from 11 mg of starting denatured protein held in the refolding reservoir. However, when 11 mg of denatured protein was loaded on a Q-sepharose column, the refolding yield and recovery of BSA would decrease to ∼40% and ∼30%, respectively, as calculated according to reported data “Refolding yield and total protein recovery as a function of the total amount of denatured-reduced BSA loaded on the column” in the literature [10]. The secondary structure of refolded BSA was determined using circular dichroism spectrometry as shown in Figure 5D, which suggested that the spectra of both column and continuous dialysis refolded protein were similar in the main CD features of secondary structures, whereas RP-HPLC analysis showed differences in the hydrophilic surfaces of protein tertiary structures.

**Figure 6:**
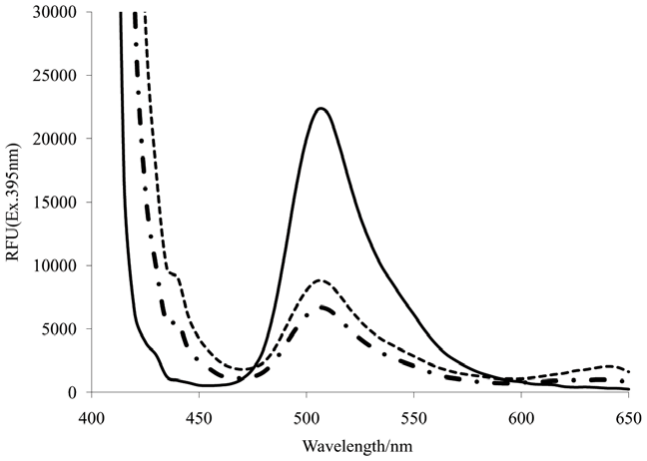
Fluorescence profiles of native EGFP (solid line), refolded EGFP by artificial chaperone-assisted refolding/continuous dialysis combination (dotted line), and by artificial chaperone-assisted refolding/simple dilution combination (dot dash line), respectively. Fluorescence profile of unfolded EGFP (negative control) was completely overlapped with x-axis.

In the refolding of EGFP, inclusion bodies were solubilized using N-lauroylsarcosine; then cyclodextrin, in the refolding buffer stripped the N-lauroylsaccosine from the target proteins, allowing the proteins to refold. A comparison of the refolding recovery of EGFP between both refolding processes is shown in Figure 6. In the simple dilution, denatured EGFP was directly diluted into a refolding buffer containing β-cyclodextrin and incubated over 8 h. The refolding yield and recovery of EGFP after 8-h incubation using artificial chaperone-assisted refolding and simple dilution combination was ∼80% and ∼37%, respectively. In the continuous dialysis, the feeding buffer containing β-cyclodextrin was added into denatured EGFP, and simultaneously striped denaturants were removed using ultrafiltration membrane. The refolding yield and recovery of EGFP using artificial chaperone-assisted refolding and continuous dialysis over 8 h was ∼80% and ∼50%, respectively.

## Discussion

To obtain active proteins from inclusion bodies for use in both biotechnology and academia is time consuming and laborious, due to the variability of the behavior of each protein that requires a great deal of trial-and-error prior to success. Currently, researchers manually test different refolding techniques and adjust various refolding conditions or parameters prior to obtaining active proteins. In this study, many refolding procedures were carried out with this apparatus, including simple dilution, dialysis, and refolding on-column. In addition, large-scale refolded protein solution achieved in the simple dilution or dialysis could be on-line concentrated or captured/purified by column. In the design of the fluid path for the refolding apparatus, the FlowDir valve (V6) was the key to integrating the dilution unit, dialysis unit and on-column unit within one apparatus. Therefore, we could combine two or three of these units together to complement each other in order to developing protein refolding techniques and processes. In this study of refolding methods, we carried out a combination of dilution and dialysis for SDF-1/CXCL12 and Trx-ARTN, dilution refolding for Trx-IGF1 and EGFP and on-column refolding for BSA, followed by protein concentration and purification on column.

We also tried to optimize the protein refolding process by means of the descending denaturant concentration method followed by protein concentration and purification on column. A descending denaturant concentration gradient was generated using a reverse-dilution method and a continuous dialysis method. Using this strategy, the efficiency of protein refolding was found to produce a higher quantity of soluble and correctly folded protein than that of previous refolding processes. In the refolding of SDF-1/CXCL12, a combination of reverse-dilution and dialysis method produced almost double amount of soluble protein than a combination of dilution and dialysis method. Soluble and purified SDF-1/CXCL12 from both refolding processes could self-associate and had similar main CD features of secondary structures as previously reported. Although in the refolding of Trx-ARTN a combination of reverse-dilution and dialysis method could not change the protein refolding yield of soluble protein, indeed it could increase the protein refolding recovery and produce almost three-fold the amount of intermolecular disulphide bridge formed homodimer than a combination of dilution and dialysis method. In contrast with previous refolding processes, the protein refolding yields of soluble proteins of Trx-IGF1 and BSA were not improved obviously as the same to Trx-ARTN using a slowly descending denaturant concentration method, whereas the protein refolding recovery of correctly folded protein was increased. In the refolding of Trx-IGF1 using reverse-dilution method, protein aggregation was suppressed to some extent and the yield of monomer was increased. The protein aggregation in the refolding of Trx-IGF1 would be significantly suppressed again using the refolding buffer containing L-Arg as identified in the literature [28]. In the refolding of BSA, a comparison of protein refolding recovery of both column and continuous dialysis refolded protein was comparable in the hydrophilic surfaces of protein tertiary structures, and the continuous dialysis method was more suitable for recovering right-folded protein from large-scale starting amount of denatured protein. In the refolding of EGFP, detergent-cyclodextrin artificial chaperone system was investigated after 8 h refolding time. The protein refolding yield and recovery were not obviously improved using continuous dialysis method, whereas the amount of cyclodextrin decreased 3 folds. These results suggested that slowly descending denaturant concentration followed with on-column concentration or on-line purification performed by this refolding apparatus could be a useful tool for a preparative scale protein production.

The reason that the slowly descending denaturant concentration method could help protein refolding can be explained as follows. Nascent polypeptides pass through structural intermediates before the native state is reached. Folding intermediates typically expose hydrophobic residues that are normally buried in the native state. These exposed hydrophobic surfaces are prone to trigger aggregation of proteins [29]. Preventing the accumulation of aggregation-prone folding intermediates is the first and most effective way to increase the molecular flux through productive folding pathways [29], [30]. Recovering active proteins from denatured proteins also uses these same folding pathways. Under denaturing conditions, the folded polypeptide chain is destabilized by denaturants. Upon the removal of denaturants, the polypeptide chain establishes interactions with itself and the solvent from unfolded to folded states. In the simple dilution refolding technique, the accumulation of aggregation-prone folding intermediates, if no aggregation inhibitors (additives) were added, would trigger the aggregation of proteins due to rapidly reduced denaturant concentrations. The step-wise dialysis method establishes sequential protein structures in parallel with step-wise reduced denaturant concentrations. At each step-wise reduction of the denaturant concentration, the denaturants (or additives) prevent the accumulation of aggregation-prone folding intermediates. Therefore, the step-wise dialysis refolding method is widely used in the lab [31] to prevent aggregation and improve the refolding recovery yield. It has often been noted that if the accumulation of aggregation-prone intermediates was not prevented using step-wise reduced denaturant concentration methods it would trigger the aggregation of proteins. Perhaps this is the reason is that synchronization was not established between protein refolding pathways and descending denaturant concentration gradient. Therefore, slowly descending denaturant concentration is a good choice to establish synchronization with the protein refolding pathway.

In this apparatus, the change rate of concentration of denaturants (or additives) can be manipulated to control the protein folding pathway in order to improve the refolding efficiency. Other factors affecting protein folding, such as redox environment, ionic strength, and pH, would be monitored and manipulated on this refolding apparatus. This refolding apparatus system has a great potential to support multiple operations for different applications, not limited to these studies in this paper. In the refolding of MMP-12 [13], we used continuous dialysis to generate a slowly descending concentration gradient of denaturant to allow protein to refold. The change of concentration of denaturant (8 M urea) in the reservoir with time in the refolding of MMP-12 using continuous dialysis method was shown in Figure S4. It took 3 days to refold MMP-12. In other work from our group, we found that the concentration of urea from 4 M to 3 M urea was a key step in the refolding process of MMP-12 [32]. Therefore, the change rate of concentration of urea from 4 M to 3 M was set at a small value of F/V, and the protein aggregation was significantly prevented. The change of concentration of urea in the reservoir with time of improved MMP-12 refolding process was shown in Figure S4. Improved continuous dialysis process obviously reduced refolding time to 1 day. The refolding of MMP-12 began with rapidly continuous dialysis from 8 M urea to 4 M urea, and then followed with slowly continuous dialysis between 4 M urea and 0.5 M urea, finally ended with 0 M urea by rapidly continuous dialysis. Detectors used in the apparatus were just UV-detector, conductivity detector and pH detector. We also could potentially integrate more detectors within this apparatus such as redox environment detector and dynamic light scattering device (DLS). Specifically, DLS is a good means of obtaining better insights into protein behavior under different environment conditions [33], [34]. With the help of DLS, we could obtain a real-time quantitative data on aggregation kinetics to monitor protein refolding process and optimize protein refolding environment conditions. The design of real-time multi-parameter detection could help to manipulate the protein refolding process.

In conclusion, we integrated three refolding techniques into our apparatus: varying dilution, dialysis and on-column refolding. This automatic refolding apparatus can be used for convenience and as a means of developing protein refolding techniques.

## Materials and Methods

### Refolding Apparatus Assembly

Figure 1 is the schematic representation of the automated refolding apparatus. This refolding apparatus consists of a compact separation unit and a control system unit. The compact separation unit was assembled using peristaltic pumps (LongerPump), an ultrafiltration device (Millipore), two 4-channel gradient valves, five 2-way valves, two 5-way valves, a refolding reservoir with stirrer, PTFE tubing, peek tubing, tube connectors (BEIONFUID), one 6-way valve and an Amershan UPC-900 in-line monitor (GE Healthcare). All of the accessories of the control system of the Programmable Logic Controller (PLC) were purchased from Shanghai Guoqiang Bioengineering Equipment Co., LTD. A laptop computer running system control software on Windows 7 OS (Microsoft) was connected to the control system via a TCP/IP protocol.

### Denatured Proteins Preparation

The gene encoding EGFP was cloned into pTO-T7 expression vector [25], then the plasmid was transferred into BL21 (DE3) CodonPlus strain (Stratagene). The DNA region coding for the human SDF-1/CXCL12, ARTN and IGF1 was obtained by PCR amplification from cDNA sequences. The gene encoding SDF-1/CXCL12 was cloned into the *NdeI* and *Xhol* sites of pET30a expression vector (Novagen), and the genes encoding ARTN and IGF1 were cloned into the *KpnI* and *Xhol* sites of pET32m expression vector (Novagen). The plasmids were transferred into BL21 (DE3) CodonPlus strain (Stratagene). SDF-1/CXCL12, Trx-ARTN, Trx-IGF1 and EGFP were expressed in *E. coli* as inclusion bodies. The cells were grown in 1 liter of LB medium at 37°C until the absorbance at 600 nm (OD_600_) reached 0.7–0.8. At this time, isopropyl-β-D-thiogalactopyranoside (IPTG) was added to 1 mM to induce protein expression at 37°C for another 4 h. The cells were harvested by centrifugation at 6,000 g at 4°C for 20 min, and washed twice with 150 ml of cell washing buffer (20 mM Tris, pH 8.0), centrifuged (6,000 g, 4°C, 20 min). The cells were lysed with 20 ml of cell lysing buffer (50 mM TrisHCl, 1 mM EDTA, 50 mM NaCl, pH 8.0) by sonication on ice. The lysate was centrifuged (10,000 g, 4°C, 20 min). The pellets were washed twice with 150 ml of inclusion bodies washing buffer (50 mM TrisHCl, 50 mM NaCl, 0.5% Triton X-100, 2 mM β-mercaptoethanol, 2 M urea, 1 mM EDTA, pH 8.0), the solution was stirred for 1 h, and then centrifuged (10,000 g, 4°C, 20 min). To remove Triton X-100, the inclusion bodies were washed two more times with 150 ml of cell washing buffer (20 mM TrisHCl, pH 8.0) and stirred for 1 h. Finally, the pellets were kept at −20°C.

The isolated inclusion bodies (SDF-1/CXCL12, Trx-ARTN and Trx-IGF1) were dissolved in 10 ml denaturing buffer (50 mM TrisHCl, 6 M GdnHCl, 10 mM β-mercaptoethanol, pH 8.0). The solution was stirred overnight and then centrifuged (20,000 g, 4°C, 60 min). The supernatant was collected and pellets were discarded. Denatured Trx-ARTN and Trx-IGF1 were purified by Ni chelating sepharose FF (GE Healthcare) under denaturing conditions. Denaturing buffer containing 50 mM imidazole was used to wash unbound and impure proteins. The target protein was eluted with denaturing buffer containing 400 mM imidazole. The total protein concentration was measured with the Bradford assay [35], [36].

The preparation of inclusion bodies and denatured EGFP protein was described in the Merck iFold™ protein refolding screening system one kit protocol [27]. Denatured EGFP was placed in the denaturing buffer containing 0.06% (w/v) N-Lauroylsarcosine.

Pure BSA was purchased from Sigma-Aldrich (St. Louis, MO) and dissolved in a denaturing buffer containing 8 M urea as previously reported [10].

### Batch Refolding of SDF-1/CXCL12 by Dilution and Dialysis Combination Refolding Method

An animation of the entire refolding process of SDF-1/CXCL12 by the automated refolding apparatus was supplied in the supporting information (Video: CXCL12 refolding process of dilution and dialysis combination.mp4).

In the preparation stage, FlowDir valve (V6) was set to link pump (P1) and the reservoir. ddH_2_O was used to rinse four inlets (A1, A2, A3, A4) of the 4-way gradient valve (V1) through pump (P1) into the reservoir at a flow rate of 40 ml/min for 1 minute. ddH_2_O in the reservoir was pumped out through 3-way valve (V2) by pump (P2). The SDF-1/CXCL12 denaturing buffer (50 mM TrisHCl, 6 M GdnHCl, 10 mM β-mercaptoethanol, pH 8.0) and refolding buffer (50 mM TrisHCl, 0.75 M L-Arg, 5 mM reduced glutathione, 0.5 mM oxidized glutathione, pH 8.5) were used to rinse two inlets (A1, A2) of 4-way gradient valve (V1) through pump (P1) into the reservoir at a flow rate of 20 ml/min for 1 minute. Then the solution in the reservoir was pumped out through 3-way valve (V2) by pump (P2).

In the refolding and purification stage, 300 ml of refolding buffer was added into the reservoir through pump (P1) at a flow rate of 100 ml/min. Then, 30 ml of denaturing protein (A1) at 1.0 mg/ml was slowly injected into the reservoir at 1 ml/min. 2.5 ml of refolding buffer was used to push the residual denatured protein into the reservoir at 1 ml/min. After 24 h, FlowDir valve (V6) was set to link pump (P1) and DirFlow valve (V8). Equilibrium buffer (20 mM TrisHCl, 50 mM NaCl, pH 8.5) was used to rinse inlet A3 of the 4-way gradient valve (V1), pump (P1), the detector and ultrafiltration device throughout 3-way valve (V2) at a flow rate of 10 ml/min for 5 min. FlowDir valve (V6) was set to link pump (P1) and the reservoir, or the reservoir and DirFlow valve (V8). Equilibrium buffer was continuously added into the reservoir by pump (P1) at a flow rate of 2 ml/min. The outlet of the reservoir provided a steady continuous flow of fluid at 50 ml/min by pump (P2) into the ultrafiltration device. The retained solution returned to the reservoir through the detector. The flow rate of the filtrate was set at 2 ml/min. After 12 h of dialysis, FlowDir valve (V6) was set to link pump (P1) and FlowDir valve (V7), the FlowDir valve (V7) was set to link FlowDir valve (V6) and off-column way, and the detector was set within the purification system. Equilibrium buffer and eluent (20 mM TrisHCl, 1 M NaCl, pH 8.5) were used to rinse the purification system at a flow rate of 20 ml/min for 1 minute. Afterwards, FlowDir valve (V7) was set to link FlowDir valve (V6) and the CM-sepharose fast flow column (5 ml). Equilibrium buffer was used to equilibrate the column at a flow rate of 2 ml/min for 5 column volumes (CVs). FlowDir valve (V6) was set to link pump (P1) and the reservoir, or FlowDir valve (V8) and FlowDir valve (V7). Refolded SDF-1/CXCL12 in the reservoir was directly loaded onto the CM-sepharose fast flow column by pump (P2) at a flow rate of 5 ml/min. The residual sample in the reservoir and ultrafiltration device was pushed into the column with equilibrium buffer by pump (P1) at a flow rate of 2 ml/min. The column was washed with equilibrium buffer until the UV absorbance at 280 nm returned to a stable baseline. The refolded SDF-1/CXCL12 protein was eluted with a 20 CV linear gradient from 0% to 100% eluent at a flow rate of 2 ml/min.

In the final stage, ddH_2_O was used to wash the entire system. FlowDir valve (V7) was set to link FlowDir valve (V6) and the off-column way. The detector was set within the purification system and ddH_2_O (A1, A2) was used to wash the purification system at a flow rate of 20 ml/min for 1 minute. After that, FlowDir valve (V7) was set to link FlowDir valve (V6) and the column. ddH_2_O was used to wash the column. FlowDir valve (V6) was set to link pump (P1) and the reservoir. ddH_2_O was used to wash the reservoir at a flow rate of 40 ml/min for 5 min and ddH_2_O in the reservoir was pumped out through 3-way valve (V2) by pump (P2). FlowDir valve (V6) was set to link pump (P1) and DirFlow valve (V8). ddH_2_O was used to wash the ultrafiltration device throughout Out(1) at a flow rate of 40 ml/min for 5 min.

The refolded SDF-1/CXCL12 eluted from the CM-sepharose fast flow column was concentrated using an YM-3 membrane (Amicon) and purified by a Superdex-75 10/300 column (GE Healthcare) on an AKTApurifier UPC100.

Two different protein concentrations of purified SDF-1/CXCL12 (1.2 and 8 mg/ml) were analyzed by a Superdex-75 10/300 column.

### Batch Refolding of SDF-1/CXCL12 by Reverse-dilution and Dialysis Combination Refolding Method

The animation of the entire refolding process of SDF-1/CXCL12 by the automated refolding apparatus was supplied in the supporting information (Video: CXCL12 refolding process of reverse-dilution and dialysis combiantion.mp4).

The preparation stage was the same as described in the section of “Batch refolding of SDF-1/CXCL12 by dilution and dialysis combination refolding method.”

In the refolding and purification stage, 30 ml of denatured protein at 1.0 mg/ml was added into the reservoir through pump (P1) at a flow rate of 2 ml/min. Then, 300 ml of refolding buffer (A2) was slowly injected into the reservoir at a flow rate of 0.23 ml/min for approximately 21 h. The rest of the procedure was the same as described in the section of “Batch refolding of SDF-1/CXCL12 by dilution and dialysis combination refolding method”.

The refolded SDF-1/CXCL12 eluted from CM-sepharose fast flow column was concentrated using a YM-3 membrane (Amicon) and purified by a Superdex-75 10/300 column (GE Healthcare) on an AKTApurifier UPC100.

Two different protein concentrations of purified SDF-1/CXCL12 (1.6 and 8 mg/ml) in were analyzed by a Superdex-75 10/300 column.

### Batch Refolding of Trx-ARTN by Dilution and Dialysis Combination Refolding Method

The animation of the entire refolding process of Trx-ARTN by the automated refolding apparatus was the same as that of SDF-1/CXCL12.

All operations were the same as described in the section of “Batch refolding of SDF-1/CXCL12 by dilution and dialysis combination refolding method”. 45 ml of denatured protein at 0.49 mg/ml was slowly added into 450 ml of refolding buffer (50 mM TrisHCl, 240 mM NaCl, 10 mM KCl, 0.4 M sucrose, 0.75 M L-Arg, 5 mM EDTA, 5 mM reduced glutathione, 0.5 mM oxidized glutathione, pH 9.5). The equilibrium buffer was 20 mM TrisHCl, 1 mM EDTA, pH 8.0. The eluent was 20 mM TrisHCl, 1 mM EDTA, 1.0 M NaCl, pH 8.0.

The refolded Trx-ARTN eluted from the CM-sepharose fast flow column was analyzed by reduced SDS-PAGE and non-reduced SDS-PAGE.

### Batch Refolding of Trx-ARTN by Reverse-dilution and Dialysis Combination Refolding Method

The animation of the entire refolding process of Trx-ARTN by the automated refolding apparatus was the same as that of SDF-1/CXCL12.

All operations were the same as described in the section of “Batch refolding of SDF-1/CXCL12 by reverse-dilution and dialysis combination refolding method”. 370 ml of refolding buffer was slowly added into 37 ml of denatured protein at 0.64 mg/ml.

The refolded Trx-ARTN eluted from the CM-sepharose fast flow column was analyzed by reduced SDS-PAGE and non-reduced SDS-PAGE.

### Batch Refolding of Trx-IGF1 by Direct Dilution Refolding Method

The animation of the entire refolding process of Trx-IGF1 by the automated refolding apparatus was shown in the supporting information (Video: Trx-IGF1 refolding process of dilution.mp4).

The preparation stage was the same as described in the section of “Batch refolding of SDF-1/CXCL12 by dilution and dialysis combination refolding method”. The ultrafiltration device was removed from the refolding apparatus.

In the refolding and purification stage, 450 ml of refolding buffer (50 mM TrisHCl, 1 mM reduced glutathione, 0.1 mM oxidized glutathione, pH 8.0) was added into the reservoir through pump (P1) at a flow rate of 100 ml/min. Then, 43 ml of denatured protein (A1) at 0.7 mg/ml was slowly injected into the reservoir at 1 ml/min. 2.5 ml of refolding buffer was used to push the residual denaturing protein into the reservoir at 1 ml/min. After 24 h, FlowDir valve (V6) was set to link pump (P1) and DirFlow valve (V8). Equilibrium buffer (50 mM TrisHCl, pH 8.0) was used to rinse inlet A3 of the 4-way gradient valve (V1), pump (P1), detector and tubes in the dialysis unit throughout Out(1) at a flow rate of 10 ml/min for 5 min. FlowDir valve (V6) was set to link pump (P1) and FlowDir valve (V7). FlowDir valve (V7) was set to link FlowDir valve (V6) and off-column way. The detector was set within the purification system and equilibrium buffer and eluent (50 mM TrisHCl, 1.0 M imidazol, pH 8.0) were used to rinse the purification system at a flow rate of 20 ml/min for 1 min. After that, FlowDir valve (V7) was set to link FlowDir valve (V6) and the Ni-chelating fast flow column (20 ml). Equilibrium buffer was used to equilibrate the column at a flow rate of 4 ml/min for 5 column volumes (CVs). FlowDir valve (V6) was set to link pump (P1) and the reservoir, or the FlowDir valve (V8) and the FlowDir valve (V7). Refolded Trx-IGF1 in the reservoir was directly loaded onto the Ni-chelating sepharose fast flow column by pump (P2) at a flow rate of 10 ml/min. The residual sample in the reservoir and ultrafiltration device was pushed into the column with equilibrium buffer by pump (P1), at a flow rate of 4 ml/min. The column was washed with 5% eluent until the UV absorbance at 280 nm returned to a stable baseline. The refolded Trx-IGF1 protein was eluted with 50% eluent at a flow rate of 4 ml/min.

The last stage was the same as described in the section of “Batch refolding of SDF-1/CXCL12 by dilution and dialysis combination refolding method”.

The refolded Trx-IGF1 eluted from Ni-column was concentrated using a YM-10 membrane (Amicon) and purified by a Superdex-200 10/300 column. The monomeric protein fractions were collected and concentrated using a YM-10 membrane (Amicon). The concentrated protein solution was purified again with a Superdex-200 10/300 column. The monomeric and pure Trx-IGF1 was collected and analyzed by a Superdex-200 10/300 column.

### Batch Refolding of Trx-IGF1 by Reverse-dilution Refolding Method

The animation of the entire refolding process of Trx-IGF1 by the automated refolding apparatus was shown in the supporting information (Video: Trx-IGF1 refolding process of reverse-dilution.mp4).

The preparation stage was the same as described in the section of “Batch refolding of SDF-1/CXCL12 by dilution and dialysis combination refolding method”. The ultrafiltration device was removed from the refolding apparatus.

In the refolding and purification stage, 50 ml of denatured protein (A1) at 0.7 mg/ml was added into the reservoir through pump (P1) at a flow rate of 2 ml/min, and then 450 ml of refolding buffer was slowly injected into the reservoir at 0.35 ml/min. After 24 h, FlowDir valve (V6) was set to link pump (P1) and DirFlow valve (V8). Equilibrium buffer was used to rinse inlet A3 of the 4-way gradient valve (V1), pump (P1), the detector and tubes in the dialysis unit throughout Out(1) at a flow rate of 10 ml/min for 1 min. FlowDir valve (V6) was set to link pump (P1) and FlowDir valve (V7). FlowDir valve (V7) was set to link FlowDir valve (V6) and off-column way. The detector was set within the purification system, and equilibrium buffer and eluent were used to rinse the purification system at a flow rate of 20 ml/min for 1 min. After that, FlowDir valve (V7) was set to link FlowDir valve (V6) and the Ni-chelating sepharose fast flow column (20 ml). Equilibrium buffer was used to equilibrate the column at a flow rate of 4 ml/min for 5 column volumes (CVs). FlowDir valve (V6) was set to link pump (P1) and the reservoir, or the reservoir and DirFlow valve (V8). Refolded Trx-IGF1 solution was used to rinse pump (P2) and was returned to the reservoir. FlowDir valve (V6) was set to link pump (P1) and the reservoir, or the FlowDir valve (V8) and the FlowDir valve (V7). Refolded Trx-IGF1 in the reservoir was directly loaded onto the Ni-chelating sepharose fast flow column by pump (P2) at a flow rate of 10 ml/min. The residual sample in the reservoir and ultrafiltration device was pushed into the column with equilibrium buffer by pump (P1) at a flow rate of 4 ml/min. The column was washed with 5% eluent until the UV absorbance at 280 nm returned to a stable baseline. The refolded Trx-IGF1 protein was eluted with 50% eluent at a flow rate of 4 ml/min.

The last stage was the same as described in the section of “Batch refolding of SDF-1/CXCL12 by dilution/dialysis combination refolding method”.

The refolded Trx-IGF1 eluted from Ni-column was concentrated using a YM-10 membrane (Amicon) and purified using a Superdex-200 10/300 column. The monomeric protein fractions were collected and concentrated using a YM-10 membrane (Amicon). The concentrated protein solution was purified again using a Superdex-200 10/300 column. The monomeric and pure Trx-IGF1 was collected and analyzed by a Superdex-200 10/300 column.

### Batch Refolding of BSA by on-column Refolding Method

The animation of the entire refolding process of BSA by the automated refolding apparatus was shown in supporting information (video: BSA refolding process of on-column.mp4).

In the preparation stage, FlowDir valve (V7) was set to link pump (P3) and the off-column way. The detector was set within the purification system, and ddH_2_O was used to rinse four inlets (B1, B2, B3, B4) of the 4-way gradient valve (V4) through pump (P3) at a flow rate of 40 ml/min for 1 min. FlowDir valve (V7) was set to link pump (P3) and the HiTrap Q-sepharose FF column (1 ml). ddH_2_O was used to rinse the column at a flow rate of 1 ml/min for 5 CVs. FlowDir valve (V7) was set to link pump (P3) and the off-column way. Denaturing buffer (50 mM TrisHCl, 3 mM EDTA, 8 M urea, pH8.5) and refolding buffer (50 mM TrisHCl, 1 mM EDTA, 79 mM Urea, 1.1 mM oxidized glutathione, 2.2 mM reduced glutathione, pH 8.5) were used to rinse the purification system at a flow rate of 20 ml/min for 1 min. FlowDir valve (V7) was set to link pump (P3) and the HiTrap Q-sepharose FF column. Denaturing buffer was used to equilibrate the column at a flow rate of 1 ml/min for 10 CVs.

In the refolding stage, 4 ml of denatured protein (B4) at 0.5 mg/ml was injected onto the column through pump (P3) at a flow rate of 1 ml/min. Then, 2.5 ml of denaturing buffer (B4) was used to push the residual denatured protein, aided by pump (P3) onto the column. The column was washed with 5 CVs to remove unbound protein. Refolding was initiated by switching from denaturing buffer to refolding buffer over 5 CVs. Then, the flow rate was set to zero, and the protein was left on the column to incubate for up to 40 h. FlowDir valve (V7) was set to link pump (P3) and the off-column way. Equilibrium buffer and eluent were used to rinse the purification system at a flow rate of 20 ml/min for 1 min. FlowDir valve (V7) was set to link pump (P3) and the HiTrap Q-sepharose FF column. Equilibrium buffer was used to wash the column with 5 CVs. Elution was then initiated by a salt gradient. A 20 CV linear gradient from 100% TrisHCl buffer containing no NaCl to that containing 1 M NaCl was applied to elute protein from the column.

In the last stage, FlowDir valve (V7) was set to link pump (P3) and the off-column way. The detector was set within the purification system and ddH_2_O was used to wash four inlets (B1, B2, B3, and B4) of the 4-way gradient valve (V4) through pump (P3) at a flow rate of 40 ml/min for 1 min. FlowDir valve (V7) was set to link pump (P3) and the HiTrap Q-sepharose FF column. ddH_2_O was used to rinse the column at a flow rate of 1 ml/min for 5 CVs.

Protein-containing fractions were pooled and analyzed by a CN reverse-phase column (Agilent ZORBAX 300SB-CN, 4.6 mm × 25 cm) on an Agilent HP1100 using 40% acetonitrile solution at flow rate of 1.0 ml/min, to determine the concentrations of refolded and partially folded protein.

### Batch Refolding of BSA by Continuous Dialysis Refolding Method

The animation of the entire refolding process of BSA by the automated refolding apparatus was shown in supporting information (video: BSA refolding process of continuous dialysis.mp4).

The preparation stage was the same as described in the section of “Batch refolding of SDF-1/CXCL12 by dilution/dialysis combination refolding method”. The ultrafiltration device was removed from the refolding apparatus.

In the refolding and purification stage, 110 ml of denatured protein (A4) at 0.1 mg/ml was added into the reservoir through pump (P1) at a flow rate of 10 ml/min. Then, approximately 480 ml of refolding buffer (A3) was continuously added into the reservoir at a flow rate of 0.2 ml/min for approximately 40 h. The outlet of the reservoir provided a steady continuous flow of fluid at 10 ml/min by pump (P2) into the ultrafiltration device, and the retained solution returned to the reservoir through detector. The flow rate of the filtrate was set at 0.2 ml/min. The urea concentration was decreased to 0.1 mM. The following work was the same as described in the section of “Batch refolding of SDF-1/CXCL12 by dilution/dialysis combination refolding method”.

Protein-containing fractions were pooled and analyzed by RP-HPLC to determine the concentrations of refolded and partially folded proteins.

### Batch Refolding of EGFP by Direct Dilution Refolding Method

The animation of the entire refolding process of EGFP by the automated refolding apparatus was shown in supporting information (video: EGFP refolding process of dilution.mp4).

In the preparation stage, FlowDir valve (V6) was set to link pump (P1) and the reservoir. ddH_2_O was used to rinse two inlets (A3,A4) of 4-way gradient valve (V1) through pump (P1) into the reservoir at a flow rate of 20 ml/min for 1 min. ddH_2_O in the reservoir was pumped out through 3-way valve (V2) by pump (P2). EGFP denaturing buffer (50 mM TrisHCl, 1.0 mM TCEP, 0.06% N-lauroylsarcosine, pH8.0) and refolding buffer (50 mM TrisHCl, 250 mM NaCl, 12.5 mM methyl-β-D-cyclodextrin, 1.0 mM TCEP, pH8.0) were used to rinse two inlets (A3,A4) of the 4-way gradient valve (V1) through pump (P1) into the reservoir at a flow rate of 20 ml/min for 1 min. Then, the solution in the reservoir was pumped out through 3-way valve (V2) by pump (P2).

In the refolding and purification stage, 500 ml of refolding buffer was added into the reservoir through pump (P1) at a flow rate of 100 ml/min, and then 50 ml of denaturing protein containing 0.06% N-lauroylsarcosine (A4) at 1.0 mg/ml was slowly injected into the reservoir at 1 ml/min. 2.5 ml of refolding buffer was used to push the residual denaturing protein into the reservoir at 1 ml/min. After 8 h (overnight), refolded protein in the reservoir was pumped out through Out(1) by pump (P2). Residual refolded protein in the reservoir was washed out by refolding buffer.

In the last stage, ddH_2_O was used to wash the reservoir and was then pumped out by pump (P2).

### Batch Refolding of EGFP by Continuous Dialysis Refolding Method

The animation of the entire refolding process of EGFP by the automated refolding apparatus was shown in supporting information (video: EGFP refolding process of continuous dialysis.mp4).

The preparation stage was the same as described in the section of “Batch refolding of BSA by continuous dialysis refolding method”.

In the refolding and purification stage, 500 ml of denatured protein containing 0.06% N-lauroylsarcosine (A4) at 0.1 mg/ml was added into the reservoir through pump (P1) at a flow rate of 20 ml/min. Then, approximately 2000 ml of refolding buffer containing 1.0 mM methyl-β-D-cyclodextrin (A3) was continuously added into the reservoir at a flow rate of 4.2 ml/min for 8 h. The outlet of the reservoir provided a steady continuous flow of fluid at 50 ml/min by pump (P2) into the ultrafiltration device, and the retained solution returned to the reservoir through the detector. The flow rate of the filtrate was set at 4.2 ml/min. After 8 h (overnight), refolded protein in the reservoir was pumped out through Out(3) by pump (P2). Residual refolded protein in the reservoir was washed out by refolding buffer.

In the last stage, ddH_2_O was used to wash the reservoir and the ultrafiltration membrane and was then pumped out by pump (P2).

To investigate the effects on EGFP refolding using direct dilution or continuous dialysis refolding techniques, the fluorescence spectra of four EGFP samples were analyzed. These samples included unfolded EGFP (negative control), soluble expressed EGFP, and two refolded EGFP from inclusion bodies and were adjusted to concentration of 1.0 mg/ml. Excitation was set at 450 nm and emission spectra at 400–650 nm were recorded using a fluorescent spectrometer SpectraMaxM5 (Molecular Devices Inc.). Slit width was set at 2.5/1 nm.

### Circular Dichroism

Purified proteins were dialyzed against phosphate buffer (10 mM Na_2_HPO_4_, pH7.4) at 4°C. CD spectra were measured at 22°C under a nitrogen flow on J-715 spectropolarimeter (Jasco) equipped with a 1 mm path-length cuvette using phosphate buffer as a control. CD spectra were recorded from190 to 250 nm with a step size of 0.1 nm, a bandwidth of 1 nm. The photomultiplier voltage read never exceeded 600 V in the spectral regions. Each spectrum was averaged from five measurements.

## Supporting Information

Figure S1 **FlowDir valve (V6) has 5 ways used for linking pump (P1), the reservoir, FlowDir valve (V8) and FlowDir valve (V7), which could switch between four positions: (A) linking pump (P1) and the reservoir, or FlowDir valve (V8) and the reservoir; (B) linking pump (P1) and FlowDir valve (V7); (C) linking pump (P1) and FlowDir valve (V8); (D) linking FlowDir valve (V8) and FlowDir valve (V7), or pump (P1) and the reservoir.**
(TIF)

Figure S2 **FlowDir valve (V7) has 4 ways used for linking pump (P3), FlowDir valve (V6), the off-column way and the column, which could switch between three positions: (A) linking FlowDir valve (V6) and the column, or pump (P3) and the off-column way; (B) linking FlowDir valve (V6) and the off-column way; (C) linking pump (P3) and the column.**
(TIF)

Figure S3 **FlowDir valve (V8) has two switch positions: the detector connected with FlowDir valve (V6) (A) or the column (B).**
(TIF)

Figure S4 **The change of concentration of urea in the reservoir with time in the MMP-12 refolding using a continuous dialysis process (solid line) and an improved continuous dialysis process (dotted line).**
(TIF)

Figure S5 **Calibration curve of protein standards of the Superdex-75 10/300 HR column.**
(TIF)

Figure S6 **Calibration curve of protein standards of the Superdex-200 10/300 HR column.**
(TIF)

Table S1 **An overview of information of five proteins.**
(DOC)

Video S1 **CXCL12 refolding process of dilution and dialysis combination.mp4.**
(MP4)

Video S2 **CXCL12 refolding process of reverse-dilution and dialysis combiantion.mp4.**
(MP4)

Video S3 **Trx-IGF1 refolding process of dilution.mp4.**
(MP4)

Video S4 **Trx-IGF1 refolding process of reverse-dilution.mp4.**
(MP4)

Video S5 **BSA refolding process of on-column.mp4.**
(MP4)

Video S6 **BSA refolding process of continuous dialysis.mp4.**
(MP4)

Video S7 **EGFP refolding process of dilution.mp4.**
(MP4)

Video S8 **EGFP refolding process of continuous dialysis.mp4.**
(MP4)
