# Supplementary figures and images for: An Automatic Refolding Apparatus for Preparative-Scale Protein Production
Source: PLoS One. 2012 Sep 27;7(9):e45891. doi: 10.1371/journal.pone.0045891 (PMC3459974; doi:10.1371/journal.pone.0045891)

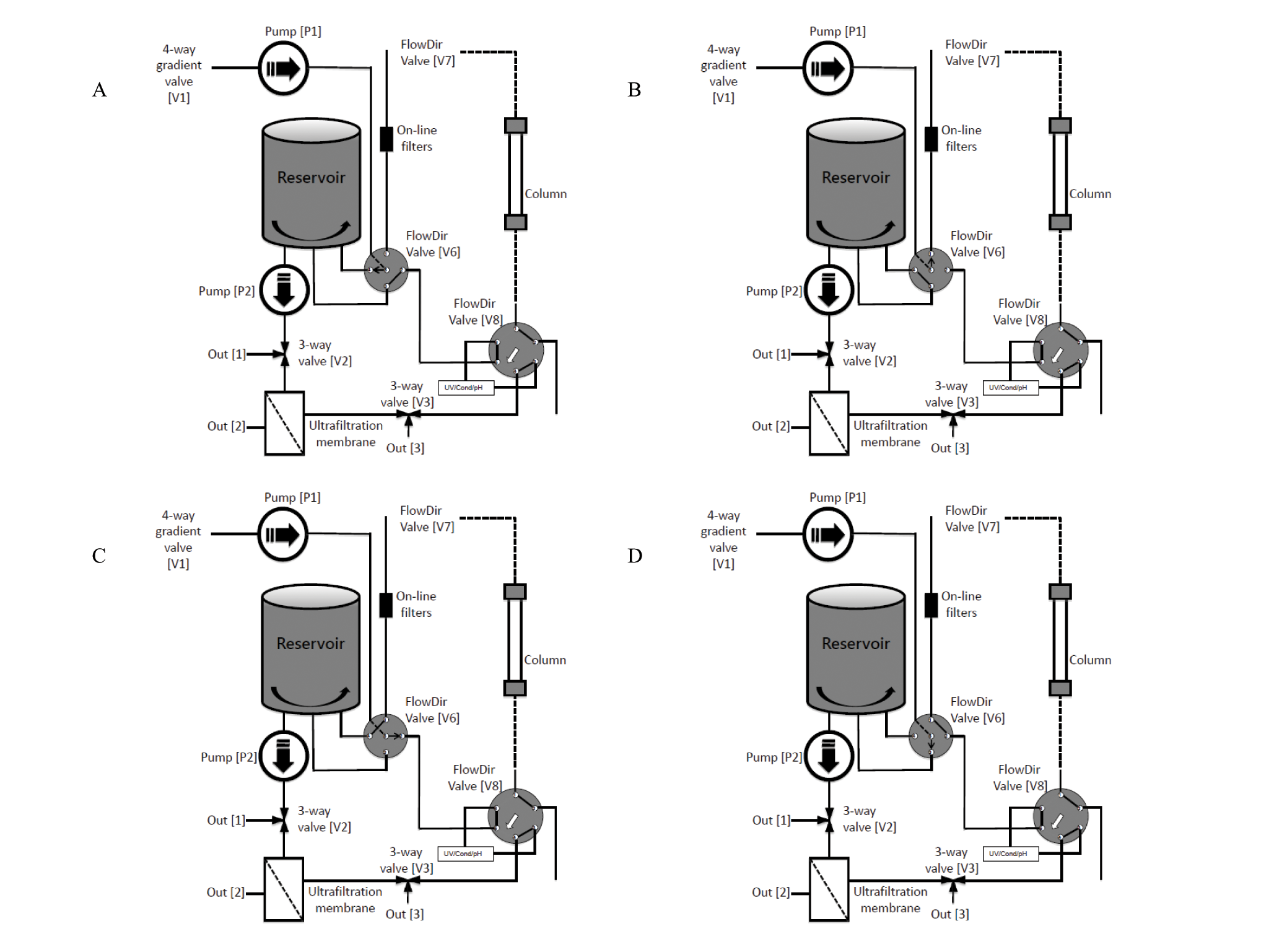

Supplement: Figure S1 — FlowDir valve (V6) has 5 ways used for linking pump (P1), the reservoir, FlowDir valve (V8) and FlowDir valve (V7), which could switch between four positions: (A) linking pump (P1) and the reservoir, or FlowDir valve (V8) and the reservoir; (B) linking pump (P1) and FlowDir valve (V7); (C) linking pump (P1) and FlowDir valve (V8); (D) linking FlowDir valve (V8) and FlowDir valve (V7), or pump (P1) and the reservoir. (TIF) [file pone.0045891.s001.tif]

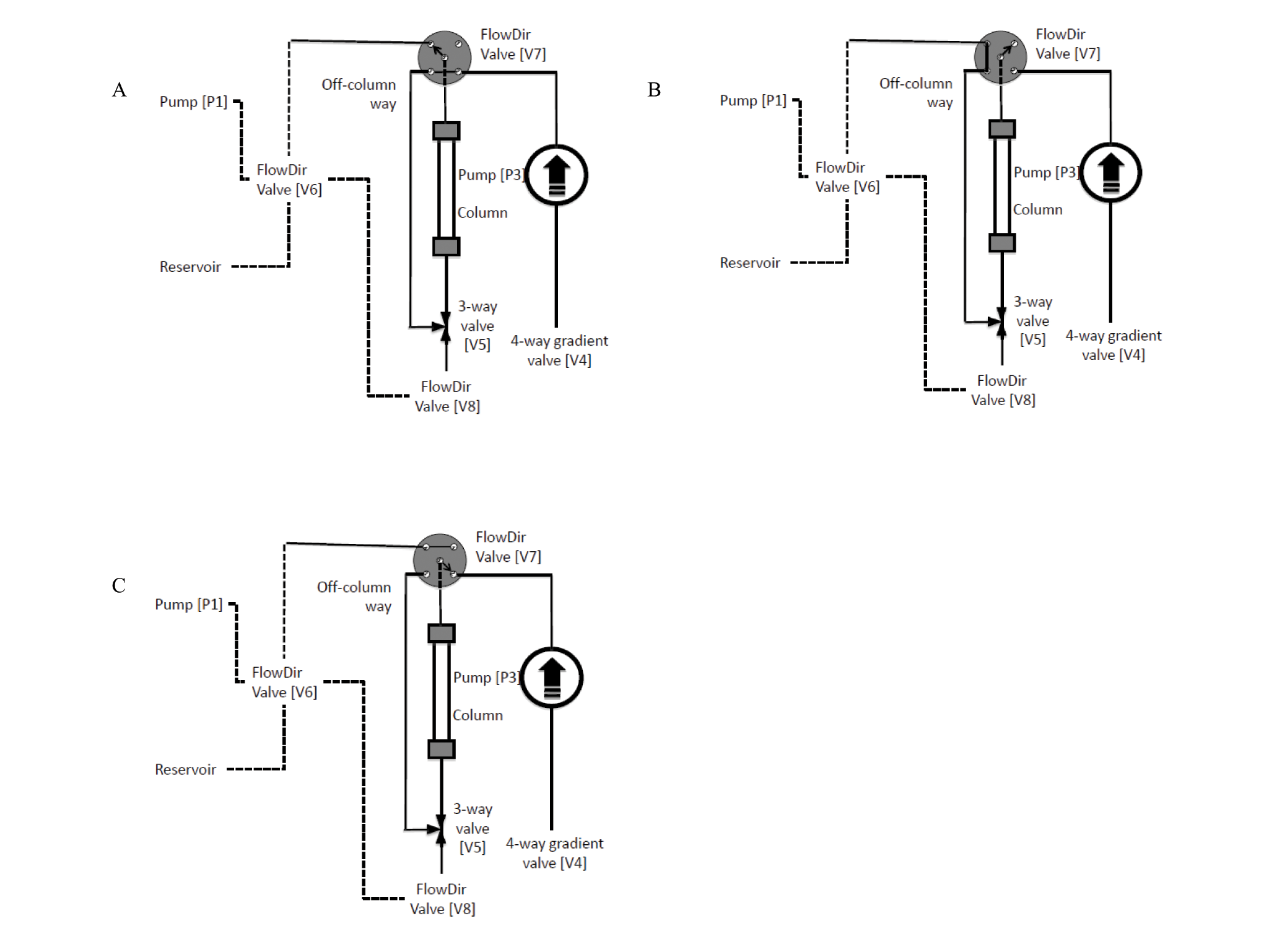

Supplement: Figure S2 — FlowDir valve (V7) has 4 ways used for linking pump (P3), FlowDir valve (V6), the off-column way and the column, which could switch between three positions: (A) linking FlowDir valve (V6) and the column, or pump (P3) and the off-column way; (B) linking FlowDir valve (V6) and the off-column way; (C) linking pump (P3) and the column. (TIF) [file pone.0045891.s002.tif]

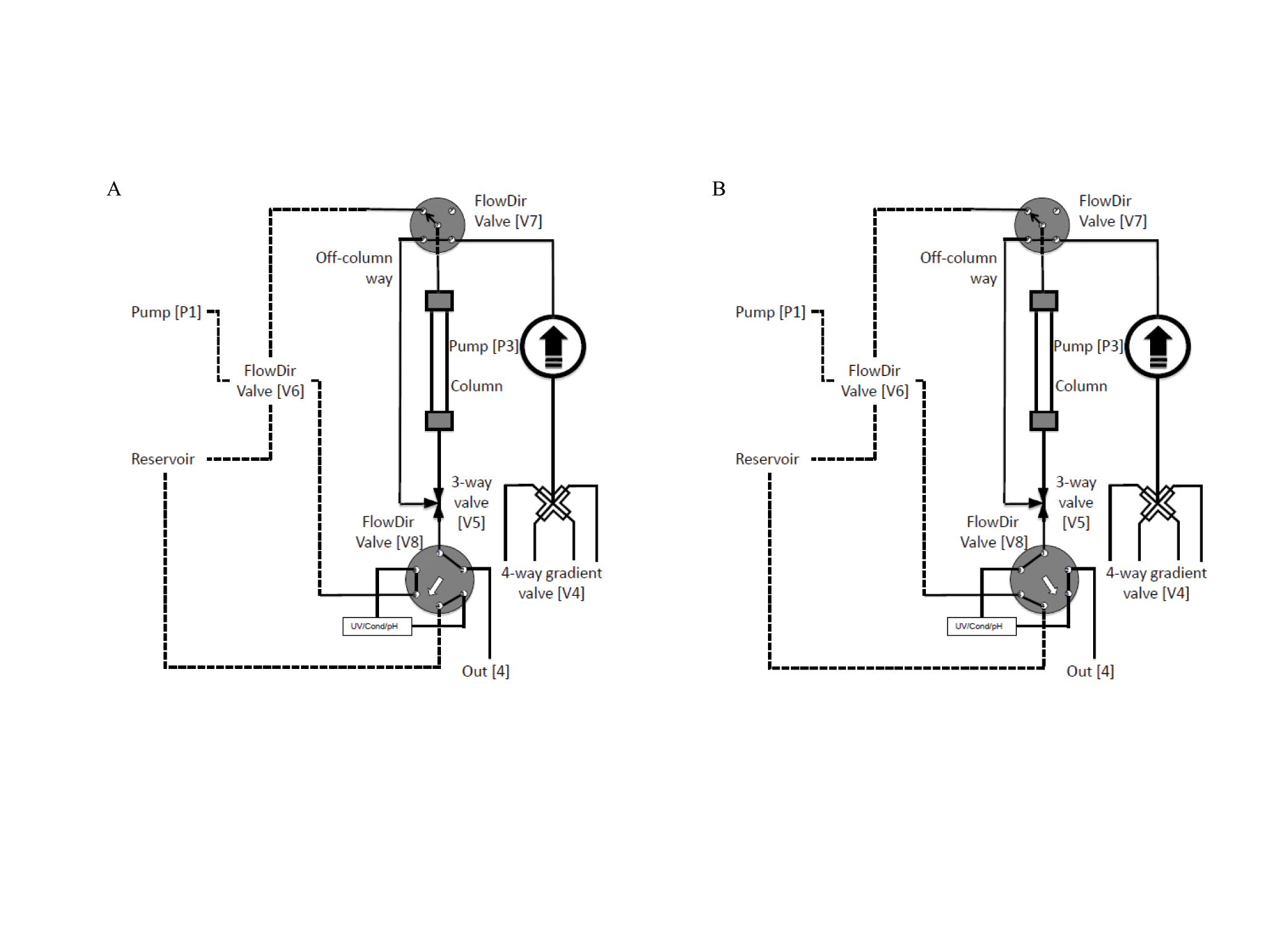

Supplement: Figure S3 — FlowDir valve (V8) has two switch positions: the detector connected with FlowDir valve (V6) (A) or the column (B). (TIF) [file pone.0045891.s003.tif]

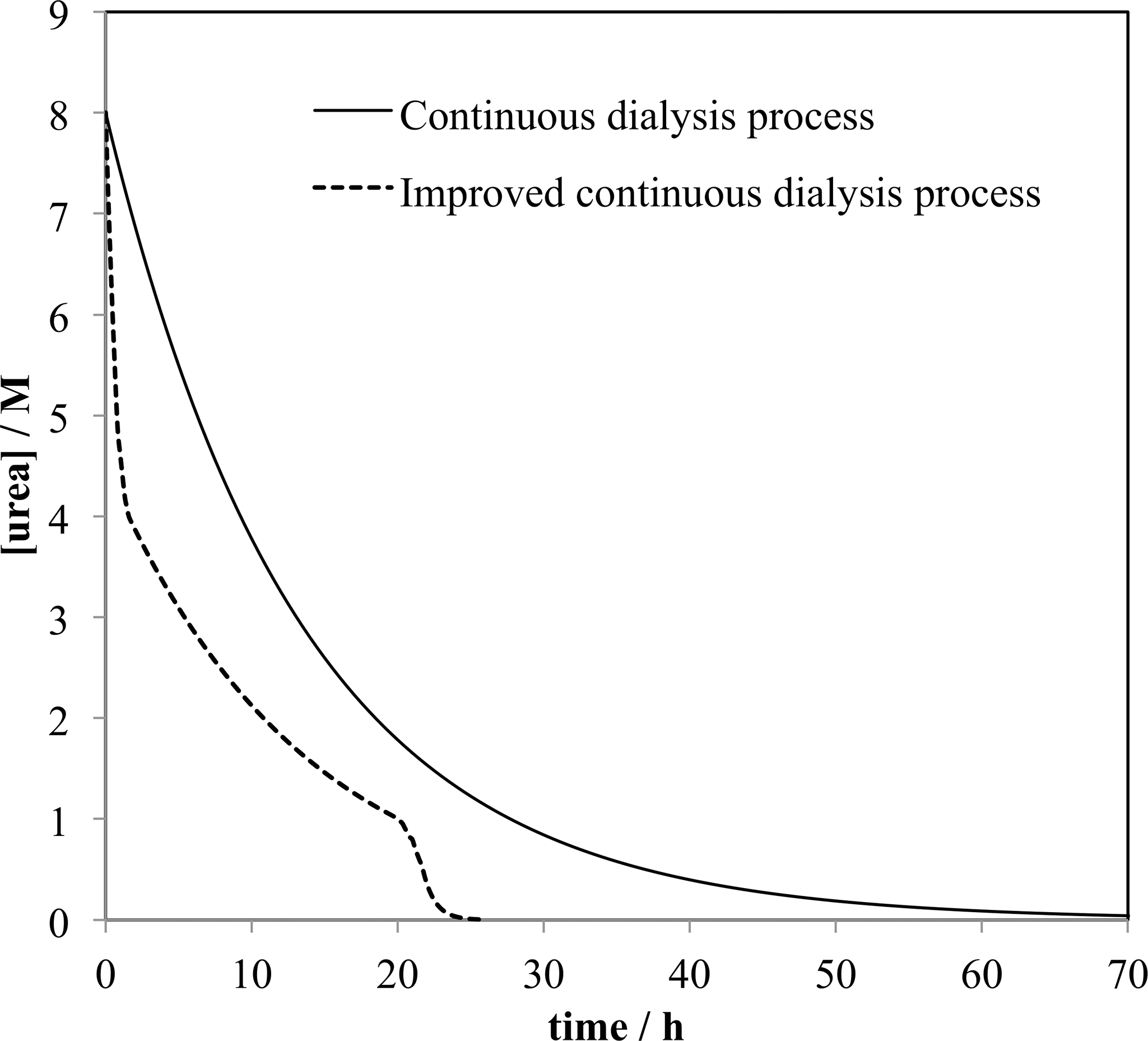

Supplement: Figure S4 — The change of concentration of urea in the reservoir with time in the MMP-12 refolding using a continuous dialysis process (solid line) and an improved continuous dialysis process (dotted line). (TIF) [file pone.0045891.s004.tif]
