# Supplementary material for: An Automatic Refolding Apparatus for Preparative-Scale Protein Production
Source: PLoS One. 2012 Sep 27;7(9):e45891. doi: 10.1371/journal.pone.0045891 (PMC3459974; doi:10.1371/journal.pone.0045891)
Supplement: Table S1 — An overview of information of five proteins. (DOC) [file pone.0045891.s007.doc]

Table S1. An overview of information of five proteins

| Proteins | Protein folding properties | | | Denaturants | Refolding | | | |
| --- | --- | --- | --- | --- | --- | --- | --- | --- |
|  | pI | MW (kDa) | Folded state |  | Previous Method | Refolding yield  and recovery | Improved Method | Refolding yield and recovery |
| SDF-1/CXCL12 | 10.5 | 8.9 | Self-associate | GdnHCl | Dilution and dialysis | ~27 %; ~84 % | Reverse-dilution and dialysis | ~54 %; ~83 % |
| Trx-ARTN | 9.0 | 26 | Dimer | GdnHCl | Dilution and dialysis | ~54 %; ~17 % | Reverse-dilution and dialysis | ~46 %; ~59 % |
| Trx-IGF1 | 5.6 | 25 | Monomer | GdnHCl | Dilution | ~90 %; ~4 % | Reverse-dilution | ~88 %; ~10 % |
| BSA | 5.6 | 66.4 | Monomer | Urea | On-column | ~60 %; ~50 % | Continuous dialysis | ~80 %; ~90 % |
| EGFP | 5.7 | 27 | Monomer | N-lauroylsaccosine | Dilution | ~80 %; ~37 % | Continuous dialysis | ~80 %; ~50 % |

Protein refolding yield was the yield of the total soluble protein compared with the starting amount of denatured proteins. Protein refolding recovery was the percentage of correctly folded protein in the total soluble protein.
